# Acute kidney injury, persistent kidney disease, and post-discharge morbidity and mortality in severe malaria in children: A prospective cohort study

**DOI:** 10.1016/j.eclinm.2022.101292

**Published:** 2022-02-12

**Authors:** Ruth Namazzi, Anthony Batte, Robert O. Opoka, Paul Bangirana, Andrew L. Schwaderer, Zachary Berrens, Dibyadyuti Datta, Michael Goings, John M. Ssenkusu, Stuart L. Goldstein, Chandy C. John, Andrea L. Conroy

**Affiliations:** a Department of Paediatrics and Child Health, Makerere University College of Health Sciences, Kampala, Uganda; b Global Health Uganda, Kampala, Uganda; c Child Health and Development Center, Makerere University College of Health Sciences, Kampala, Uganda; d Department of Psychiatry, Makerere University College of Health Sciences, Kampala, Uganda; e Department of Pediatrics, Indiana University School of Medicine, Indianapolis, IN, USA; f Ryan White Center for Pediatric Infectious Disease and Global Health, Indiana University School of Medicine, 1044 W. Walnut St., Indianapolis, IN, USA; g Department of Epidemiology and Biostatistics, Makerere University School of Public Health, Kampala, Uganda; h Cincinnati Children's Hospital, Cincinnati, OH, USA; i Center for Global Health, Indiana University School of Medicine, Indianapolis, IN, USA

**Keywords:** Acute kidney injury, Acute kidney disease, Malaria, Mortality, Cerebral malaria, Blackwater fever, Neurologic deficit, Disability, Sub-Saharan Africa, NGAL

## Abstract

**Background:**

Globally, 85% of acute kidney injury (AKI) cases occur in low-and-middle-income countries. There is limited information on persistent kidney disease (acute kidney disease [AKD]) following severe malaria-associated AKI.

**Methods:**

Between March 28, 2014, and April 18, 2017, 598 children with severe malaria and 118 community children were enrolled in a two-site prospective cohort study in Uganda and followed up for 12 months. The Kidney Disease: Improving Global Outcomes (KDIGO) criteria were used to define AKI (primary exposure) and AKD at 1-month follow-up (primary outcome). Plasma neutrophil gelatinase-associated lipocalin (NGAL) was assessed as a structural biomarker of AKI.

**Findings:**

The prevalence of AKI was 45·3% with 21·5% of children having unresolved AKI at 24 h. AKI was more common in Eastern Uganda. In-hospital mortality increased across AKI stages from 1·8% in children without AKI to 26·5% with Stage 3 AKI (*p* < 0·0001). Children with a high-risk plasma NGAL test were more likely to have unresolved AKI (OR, 7·00 95% CI 4·16 to 11·76) and die in hospital (OR, 6·02 95% CI 2·83 to 12·81). AKD prevalence was 15·6% at 1-month follow-up with most AKD occurring in Eastern Uganda. Risk factors for AKD included severe/unresolved AKI, blackwater fever, and a high-risk NGAL test (adjusted *p* < 0·05). Paracetamol use during hospitalization was associated with reduced AKD (*p* < 0·0001). Survivors with AKD post-AKI had higher post-discharge mortality (17·5%) compared with children without AKD (3·7%).

**Interpretation:**

Children with severe malaria-associated AKI are at risk of AKD and post-discharge mortality.

**Funding:**

This work was supported by the National Institutes of Health National Institute of Neurological Disorders and Stroke (R01NS055349 to CCJ) and the Fogarty International Center (D43 TW010928 to CCJ), and a Ralph W. and Grace M. Showalter Young Investigator Award to ALC.

**Research in context**

**Evidence before this study**

We searched PubMed with the search string (“acute kidney injury”) AND (“acute kidney disease”) AND (“children” OR “pediatric”) up to Sept 23, 2021 for reports in any language. The majority of acute kidney injury (AKI) occurs in low-and-middle-income countries (LMIC) where 85% of global AKI cases occur. Malaria is a leading cause of AKI in sub-Saharan Africa. Among survivors, AKI is a risk factor for persistent kidney injury leading to a post-AKI phenomenon of acute kidney disease (AKD). AKD is kidney disease of less than 90 days in duration that, if it persists, leads to chronic kidney disease (CKD). The frequency of post-AKI AKD following severe malaria in pediatric populations from LMIC has not been systematically assessed.

**Added value of this study**

To our knowledge, this is the largest study, and the first cohort study to evaluate AKD in pediatric severe malaria survivors with AKI. We report an AKI prevalence of 45% in the first 24 h of hospitalization with AKD occurring in 16% of survivors at 1-month follow-up. Children with evidence of tubular injury on admission were more likely to have unresolved AKI at 24 h and had a higher risk of AKD. AKD was more common in eastern Uganda with risk factors including severe AKI and blackwater fever on admission. Paracetamol administration in-hospital was associated with reduced risk of AKD at 1-month follow-up. Children with both AKI and AKD had a substantially higher risk of post-discharge mortality.

**Implications of all the available evidence**

This study confirms that severe malaria survivors are vulnerable to persistent kidney disease and increased mortality. Given the long-term morbidity and mortality associated with the AKI to CKD progression, an emphasis on early diagnosis and treatment of AKI is needed to prevent disease progression and promote kidney recovery. Future studies should assess the potential kidney protective action of paracetamol to prevent persistent kidney disease related to severe malaria.

Alt-text: Unlabelled box

## Introduction

In low-and-middle-income countries (LMIC), there are an estimated 18 million acute kidney injury (AKI) cases annually, representing 85% of global cases.^1^ In contrast to high-income countries, AKI in LMIC is more often community-acquired and occurs in previously healthy children.^2^ AKI is a risk factor for mortality, new disabilities at discharge, cognitive and behavioral problems, and chronic kidney disease (CKD) in surviving children.^3^, ^4^, ^5^, ^6^, ^7^ Although AKI is increasingly recognized in children with severe malaria (reviewed in^4^), the burden of malaria-associated AKI in LMIC and its impact on lifelong health outcomes, including CKD, remains poorly understood.

AKI and CKD are interconnected syndromes linked by a transitional period called acute kidney disease (AKD).^8^ AKD represents acute or subacute damage or loss of kidney function between 7 and 90 days after exposure to an AKI initiating event.^8^ Conceptually, AKD represents persistent kidney injury in the post-AKI period and offers an opportunity to intervene and positively modify long-term outcomes associated with CKD.^8^ Risk factors for AKD include severe, persistent, or relapsing AKI. Additional tools to stratify patients at risk of persistent AKI, AKD, and CKD include markers of structural kidney injury, including neutrophil gelatinase-associated lipocalin (NGAL).^8^, ^9^, ^10^ Because of the rising global burden of CKD and its associated strain on health systems,^11^ as well as the historical under-representation of sub-Saharan African in global AKI research,^3^ there is a critical need to define AKI recovery and predictors of AKD in malaria.

We hypothesize that children with malaria-associated AKI will have kidney dysfunction persist as AKD. To test this hypothesis, we defined the epidemiology of community-acquired AKI, AKI recovery, and AKD in a prospective multi-site observational study of Ugandan children with severe malaria followed for one year. We evaluated risk factors for AKI and AKD to define the impact of persistent kidney disease on morbidity and mortality in the context of severe malaria.

## Methods

### Setting

**Figure 1:**
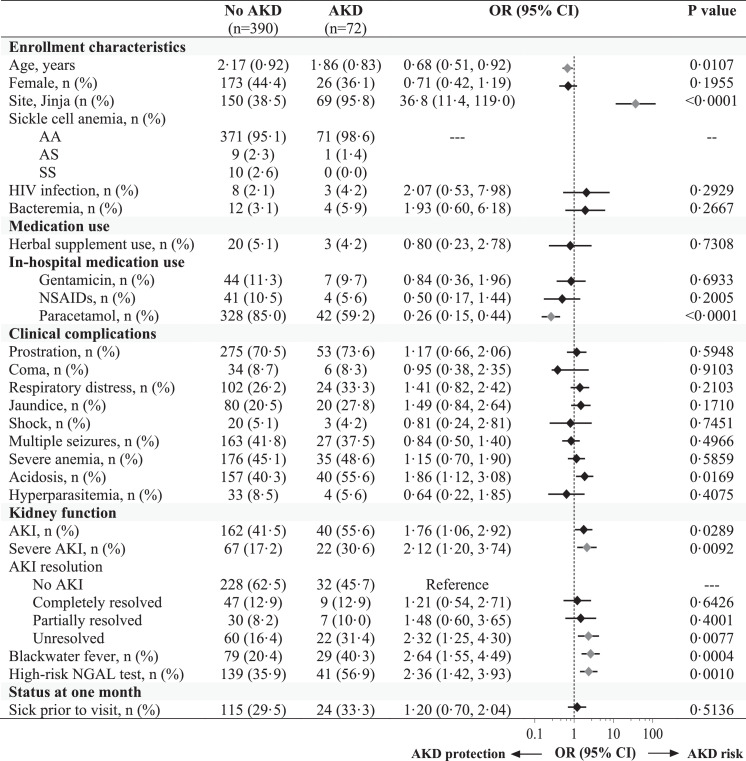
*Study flow diagram*. During hospitalization, 598 children with severe malaria and at least one creatinine measure available had acute kidney injury (AKI) defined using the Kidney Disease: Improving Global Outcomes (KDIGO) criteria based on a 1.5 fold increase in creatinine from estimated baseline or a 0.3 mg/dL change in creatinine over the first 24 h of hospitalization. AKI was stratified into stage 1 AKI or severe AKI (Stage 2 or Stage 3). Among survivors with a repeat creatinine value at 1-month follow-up we defined acute kidney disease (AKD) which reflects a 1.5-fold increase in creatinine from the estimated 1-month baseline creatinine or an estimated glomerular filtration rate < 90 mL/min per 1.73m^2^ using the Bedside Schwartz equation. Kidney disease was defined as a 1.5-fold increase in creatinine from the estimated 1-month baseline creatinine or an estimated glomerular filtration rate < 90 mL/min per 1.73m^2^ using the Bedside Schwartz equation in the community children. Children were recruited at Mulago National Referral Hospital in Kampala, Central Uganda and Jinja Regional Referral Hospital in Jinja, Eastern Uganda.

Children with severe malaria or community children were recruited from Mulago National Referral and Teaching Hospital (MNRH) in Kampala in Central Uganda and Jinja Regional Referral Hospital (JRRH) in Eastern Uganda (Figure 1). MNRH is the main National Referral Hospital for the country, located in the Kampala metropolitan area. JRRH is located 82 km east of MNRH in the Busoga sub-region on the shores of Lake Victoria and serves a catchment area of nearly two million people.

### Study design

Between March 28th, 2014 and April 18th, 2017, 600 children with severe malaria and 120 community children were enrolled in a prospective cohort study at two sites in Uganda (MNRH, JRRH) to evaluate the impact of the five most common forms of severe malaria on neurocognition at 12-months follow-up in children <5 years of age. The present study was nested within the parent study to assess the impact of AKI on kidney recovery and survival and included 598 children with severe malaria and 118 community children (Figure 1). The primary exposure was acute kidney injury, and the primary outcome was kidney recovery at 1-month follow-up (AKD). Secondary exposures included AKI severity, persistent AKI, and a high-risk NGAL test. Secondary outcomes included in-hospital mortality, duration of hospitalization, the presence of neurologic deficits at discharge, and post-discharge 12-month mortality. Children were followed for one year and were asked to return to the hospital when sick. Children with severe malaria returned for an interim health assessment and blood draw to define AKD at 1-month follow-up and vital status was assessed at 12-months follow-up. No blood draws were conducted at the 12-month visit.

A power calculation was conducted assuming a mortality rate of 8%,^12^ 95% retention at 1-month follow-up, and varied the AKI prevalence from 35 to 50%. At a fixed alpha of 0·05, we had between 83 and 87% power to detect a two-fold increase in AKD in children with AKI during hospitalization assuming a baseline AKD prevalence of 10% in children without AKI.

### Participants

Children presenting to hospital with a history of fever were screened by study teams working at the participating sites seven days a week, 24 h a day. Inclusion criteria included children aged 6 months to 4 years with malaria (positive rapid diagnostic test for *Plasmodium falciparum* histidine-rich protein-2 (HRP-2) or direct visualization of parasites by Giemsa microscopy) who required hospitalization for prostration; severe anemia, hemoglobin< 5 g/dL; two or more seizures in 24 h; respiratory distress; or coma.^13^ The definition of malaria using either rapid diagnostic test or microscopy aligns with the Uganda Clinical Guidelines on malaria diagnosis. Exclusion criteria included known chronic illness requiring medical care, known developmental delay, or history of coma, head trauma, or prior hospitalization for malnutrition. None of the children had a known history of kidney disease. Comatose children with lumbar puncture findings suggestive of another cause of coma were excluded. For controls, 120 community children from the nuclear family, extended family, or household area of the children with severe malaria were enrolled and used to assess the prevalence of kidney disease in the population and validate approaches to estimate baseline creatinine. Additional exclusion criteria for the controls included an illness requiring medical care within the previous four weeks, a major medical or neurologic abnormality at screening physical examination, or active illness.

Elevated blood urea nitrogen (BUN) was defined as a BUN ≥ 20 mg/dL^13^. Blackwater fever was defined based on parental report of tea or ‘coca-cola’ coloured urine, which has a reported agreement of 80% with urine dipsticks for hemoglobin.^6^ Shock was defined as a capillary refill time > 3 s or a lower limb temperature gradient.

### Study procedures

On enrollment, children had a complete history and physical exam, and venous blood draw. Children were managed according to Ugandan National Treatment guidelines for severe malaria, including intravenous artesunate followed by oral artemisinin-combination therapy. Fluids were administered as clinically indicated based on the treating clinician's assessment of volume status using the Ugandan Clinical Treatment guidelines. Children with dehydration based on clinical assessment of signs including slow skin pinch, dry mucous membranes, or eagerness to drink received 75 mL/kg of oral rehydration solution over 4 h orally or via a nasogastric tube. In severe dehydration, infants received 30 mL/kg of intravenous ringer's lactate or normal saline administered over one hour, and an additional 70 mL/kg administered over 5 h. Children >1 year of age received 30 mL/kg over 30 min and the next 70 mL/kg over 2·5 h. Maintenance fluids were calculated at 100 mL/kg of normal saline for the first 10 kg, plus 50 mL/kg for the next 10 kg and 25 mL/kg for the next 10 kg. Antibiotics were administered to children when indicated based on the admitting clinician's decision. A pediatric nephrologist was consulted for the clinical management of AKI. Hemodialysis was available as a fee-based service on adult wards of a nearby government-supported Kampala hospital on a limited basis.

### Laboratory evaluations

BUN, glucose, sodium, potassium, chloride, bicarbonate, and blood gasses were assessed by i-STAT (Abbott Point of Care Inc., Princeton, NJ). Serum creatinine (SCr), lactate dehydrogenase, aspartate transaminase (AST), alanine aminotransferase (ALT), and total bilirubin were tested in batches by the Indiana University Health Pathology Laboratory, accredited by The College of American Pathologists. Cryopreserved samples were stored at –80 °C until shipment to the US on dry ice. Creatinine was tested using the modified Jaffe colorimetric method traceable to an isotope dilution mass spectrometry (IDMS) reference method. To assess for bacteremia, 1–3 mL of whole blood was inoculated into pediatric blood culture bottles (Peds Plus/F) and incubated in a BACTEC 9050 Blood culture system for up to five days. Positive samples were Gram-stained and sub-cultured on blood agar, chocolate agar, or MacConkey agar plates. Malaria parasite biomass was assessed by quantifying plasma histidine-rich protein 2 (PfHRP-2) levels using a commercial enzyme-linked immunosorbent assay (ELISA) at a 1:2400 dilution (assay range, 64·8–3300 ng/mL) with a coefficient of variation of 4·2% (Malaria Ag Cellabs, Brookvale, Australia). Tubular injury was assessed by measuring plasma NGAL using a custom Luminex MagPix assay (R&D Systems, Minneapolis, MN) and categorized using established risk thresholds.^14^

### Assessment of acute kidney injury and acute kidney disease

Acute kidney injury (AKI) was defined using the Kidney Disease: Improving Global Outcomes (KDIGO) criteria based on a 1·5-fold increase in creatinine from baseline or a 0·3 mg/dL increase in creatinine within 48 h.^15^ Urine output was not assessed as it is not a standard clinical practice in Uganda. AKI was staged as follows: stage 1, 1·5–1·9-fold increase in SCr over baseline; stage 2, 2·0–2·9-fold increase over baseline; stage 3, ≥ 3·0-fold increase over baseline. AKI was classified as severe if it was stage 2 or 3^3^. Baseline SCr was estimated using a height-independent approach assuming a glomerular filtration rate (GFR) of 120 mL/min per 1·73m^2^ as described.^16^ The height-independent approach to estimate baseline SCr was validated as the best approach to estimate baseline creatinine using two independent cohorts of Ugandan community children and outperformed other approaches.^16^ SCr was measured on admission, at 24 h, and 1-month follow-up. AKI was defined as incident if AKI was not present on admission and was diagnosed at 24 h. AKI recovery was assessed at 24 h and was defined as unrecovered if AKI was still present, partial if SCr was within 50% of estimated baseline SCr, or complete if the SCr returned to within 15% of estimated baseline SCr.

Acute kidney disease (AKD) was defined in children with severe malaria if there was a 1·5-fold increase in creatinine over estimated baseline or an eGFR< 90 mL/min per 1·73m^2^ at 1-month follow-up. The same definition was applied to community children and classified as kidney disease.

### Statistical analysis

Data were analyzed using STATA v14·0 (StataCorp). Differences in continuous measures were assessed using Student's *t*-test or Wilcoxon rank-sum test, as appropriate, and differences in frequencies were assessed using Pearson's Chi-squared test or Fisher's exact test. Analyses were conducted on all participants with AKI and AKD assessed as outlined in Figure 1. Data on age, sex, and site were complete for all participants. To evaluate differences in continuous variables across AKI stages, a non-parametric test of trend or Pearson's Chi-squared test was used, as appropriate. Bivariate logistic regression was used to assess risk factors for AKI and AKD. When evaluating clinical variables associated with AKI, multiple logistic regression models were used adjusting for participant age, sex, and site. Linear regression was used to evaluate the relationship between AKI and the duration of hospitalization with the duration of hospitalization as the dependent variable and AKI as the predictor adjusting for age, sex, and site. Logistic regression was used to evaluate the relationship between AKI and neurologic deficits as well as high-risk NGAL tests and AKI, AKI resolution and mortality. To identify risk factors for AKD at 1-month follow-up, logistic regression was used. The Benjamini-Hochberg False Discovery procedure was used to adjust for multiple comparisons and applied at a threshold of 0·05, as indicated in Figure and Table legends. An adjusted p-value <0·05 was considered significant.

### Ethics

Written informed consent was obtained from the parents or legal guardians of study participants after providing emergency clinical care to stabilize children. Ethical approval was granted by Institutional Review Boards at Makerere University School of Medicine, the University of Minnesota, and Indiana University School of Medicine. The Uganda National Council for Science and Technology approved the study.

### Role of the funding source

The funder had no role in the study design, data collection, analysis, or interpretation; patient recruitment, the writing of the manuscript or the decision to submit it for publication. All authors confirm they had full access to the study data and accept responsibility to submit the publication and have approved the final version of the manuscript.

## Results

598 children with severe malaria and 118 community children were enrolled in the study (Figure 1). The mean (SD) age of all study participants was 2·1 (0·9) years with 44·1% of children female. Enrollment across sites was comparable with 44·7% of children enrolled from Jinja and 55·3% from Kampala. The mean (SD) duration of follow-up for participants was 0·86 (0·25) person-years and was longer in community children 0·94 (0·06) compared to children with severe malaria 0·84 (0·27) (not shown). Among children with severe malaria, there were no differences in parasite density or circulating parasite biomass (plasma PfHRP-2) in children based on site (*p*>0·05 for both, Supplementary Table S1). The prevalence of culture positive bacterial infections was infrequent at 3·8% and was comparable between sites (3·7% Kampala vs. 3·9% Jinja) (Supplementary Table S1).

### AKI prevalence, risk factors and recovery over hospitalization

The prevalence of AKI over hospitalization was 45·3%, with most AKI being present on hospital admission. AKI was more common and more likely to be severe in children enrolled in Eastern Uganda (Jinja) compared to Central Uganda (Kampala) (*p* < 0·001) (Supplementary Table S2). Herbal medication use was more commonly reported in Jinja (6·7%) than Kampala (2·7%) and was more frequent in children with AKI (Table 1, Supplementary Tables S1 and S3). A shorter duration of fever, herbal medication use, and the presence of shock, coma, retinopathy, respiratory distress, prostration, jaundice, and a history of blackwater fever and vomiting on admission adjusting for age, sex, and site were associated with AKI (adjusted *p* < 0·05) (Table 1). Site stratified differences in variables associated with AKI are presented in Supplementary Table S3. Among children with severe AKI on admission (AKI stage 2 or stage 3), 84·0% was unresolved at 24 h, 7·4% was partially resolved and 8·6% had resolved completely (not shown). Children from Jinja (25·9%) were more likely to have unresolved AKI compared to children from Kampala (16·8%) (Supplementary Table S2, *p* < 0·001).

**Table 1:** Demographic and clinical variables associated with AKI over hospitalization.

	No AKI (*n* = 327)	AKI (*n* = 271)	OR (95% CI)	P value	aOR (95% CI)	P value
Demographic Characteristics						
Age, years	2·2 (0·9)	2·1 (0·9)	0·89 (0·75, 1·06)	0·2063		
Female, n (%)	143 (43·7)	118 (43·5)	0·99 (0·72, 1·37)	0·9631		
Weight-for-age z score	−1·1 (1·1)	−1·0 (1·1)	1·05 (0·90, 1·23)	0·5091		
Height-for-age z score	−1·2 (1·3)	−1·0 (1·3)	1·12 (0·99, 1·27)	0·0677		
Weight-for-height z score	−0·6 (1·1)	−0·7 (1·2)	0·95 (0·82, 1·09)	0·4398		
Duration of fever, days	4·0 (2·7)	3·1 (1·7)	0·82 (0·76, 0·90)	<0·0001	0·85 (0·78, 0·92)	0·0001*
Sickle cell anemia, n (%)						
HbAA	311 (95·1)	260 (95·9)	Reference	—		
HbAS	6 (1·8)	7 (2·6)	1·40 (0·46, 4·20)	0·5537		
HbSS	10 (3·1)	4 (1·5)	0·48 (0·15, 1·54)	0·2173		
Jinja site, n (%)	114 (34·9)	154 (56·8)	2·46 (1·77, 3·42)	<0·0001	2·43 (1·74, 3·39)	<0·0001*
Medication History						
Anti-malarial, n (%)	198 (62·1)	166 (61·7)	0·98 (0·71, 1·38)	0·9289		
Artesunate, n (%)	53 (16·6)	38 (14·1)	0·83 (0·53, 1·30)	0·4064		
Antibiotic, n (%)	100 (31·4)	103 (38·3)	1·36 (0·97, 1·91)	0·0781		
Gentamicin, n (%)	13 (4·1)	11 (4·1)	1·00 (0·44, 2·28)	0·9932		
NSAIDs, n (%)	33 (10·1)	19 (7·0)	0·67 (0·37, 1·21)	0·1855		
Herbal medication use, n (%)	7 (2·1)	20 (7·4)	3·64 (1·52, 8·75)	0·0038	3·10 (1·27, 7·59)	0·0132*
Admission characteristics						
Temperature, °C	37·7 (1·2)	37·8 (1·3)	1·04 (0·91, 1·19)	0·5420		
Tachycardia	175 (53·5)	165 (60·9)	1·35 (0·98, 1·87)	0·0704		
Tachypnea	220 (67·3)	207 (76·4)	1·57 (1·09, 2·26)	0·0145	1·56 (1·07, 2·27)	0·0201
Shock, n (%)	18 (5·6)	31 (11·7)	2·24 (1·23, 4·11)	0·0088	2·38 (1·28, 4·43)	0·0061*
Coma, n (%)	29 (8·9)	44 (16·2)	1·99 (1·20, 3·27)	0·0071	1·90 (1·14, 3·16)	0·0143*
Multiple seizures, n (%)	143 (43·7)	108 (39·9)	0·85 (0·61, 1·18)	0·3389		
Retinopathy, n (%)						
No	236 (72·2)	141 (52·0)	Reference	—		
Yes	24 (7·3)	38 (14·0)	2·65 (1·53, 4·60)	0·0005	3·26 (1·84, 5·77)	<0·0001*
Not assessed	67 (20·5)	92 (34·0)	2·30 (1·58, 3·35)	<0·0001	1·79 (1·20, 2·66)	0·0040*
Respiratory distress, n (%)	56 (17·1)	119 (43·9)	3·79 (2·60, 5·51)	<0·0001	3·34 (2·27, 4·92)	<0·0001*
Prostration, n (%)	207 (63·3)	222 (81·9)	2·63 (1·79, 3·85)	<0·0001	2·48 (1·68, 3·67)	<0·0001*
Jaundice, n (%)	58 (17·7)	79 (29·3)	1·92 (1·30, 2·82)	0·0009	2·04 (1·36, 3·07)	0·0006*
Blackwater fever, n (%)	56 (17·2)	85 (31·5)	2·21 (1·50, 3·25)	0·0001	2·06 (1·37, 3·09)	0·0005*
Severe anemia, n (%)	132 (40·5)	112 (41·6)	1·05 (0·76, 1·46)	0·7775		
Vomiting, n (%)	153 (46·8)	162 (59·8)	1·69 (1·22, 2·34)	0·0016	1·61 (1·15, 2·24)	0·0053*
Diarrhea, n (%)	57 (17·4)	58 (21·4)	1·29 (0·86, 1·94)	0·2207		
Comorbid diagnoses						
Gastroenteritis, n (%)	43 (13·2)	45 (16·6)	1·31 (0·83, 2·05)	0·2484		
Pneumonia, n (%)	19 (5·9)	25 (9·2)	1·64 (0·88, 3·04)	0·1192		

Data presented as mean (SD) or n (%).

Adjusted odds ratio (aOR) calculated using logistic regression adjusting for participant age, sex, and site.

Adjusted estimates significant following adjustment for 13 multiple comparisons are indicated with an *.

**Figure 2:**
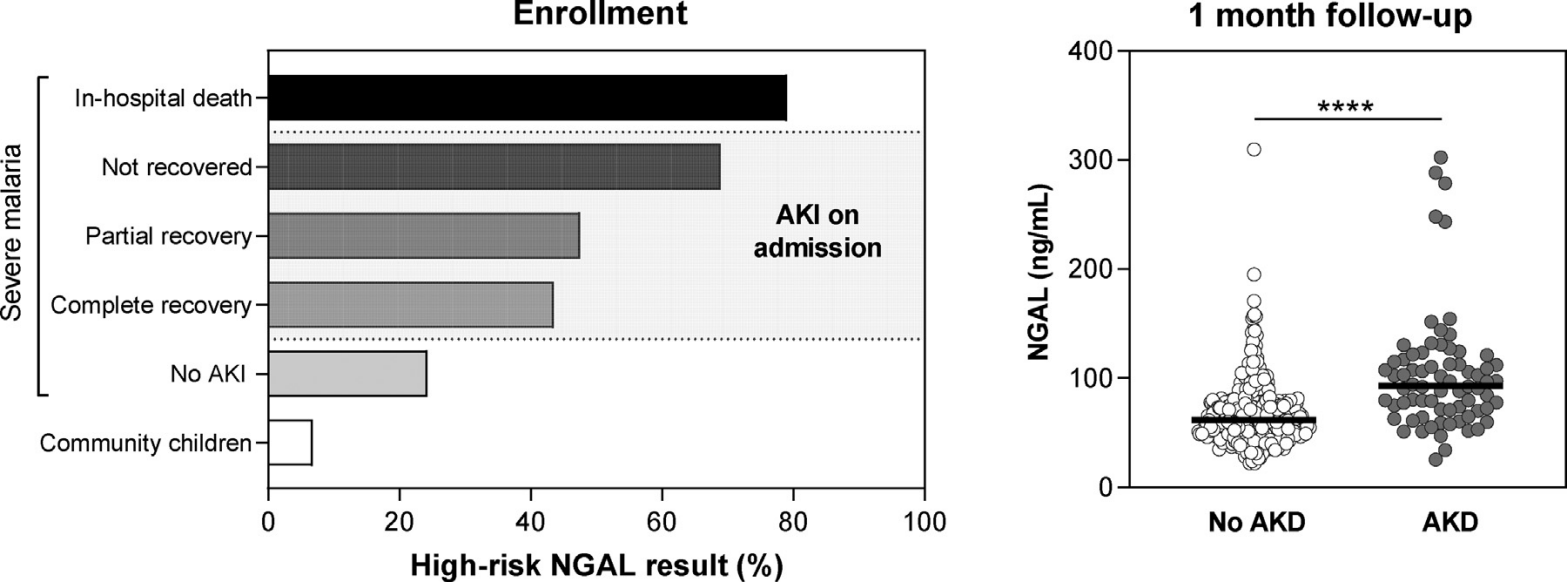
Proximal tubular injury is more common in children with persistent AKI or fatal outcomes and is elevated in children with AKD at 1-month follow-up. (Left) Bar graph depicting the frequency of a high-risk NGAL test result (NGAL ≥ 300 ng/mL) on admission in community children, children with severe malaria without AKI over hospitalization, children with AKI separated by AKI recovery status at 24 h (in gray shaded area), and children who died in-hospital. (Right) Scatter plot and median depicting NGAL levels at 1-month follow-up in survivors based on the presence of AKD. Analysis by non-parametric Wilcoxon rank sum test where **** adjusted *p* < 0·0001.

Laboratory findings associated with AKI and AKI severity included elevated BUN, hyperkalemia, hyponatremia, hypoglycemia, elevated lactate dehydrogenase, total bilirubin, AST, and ALT as well as acidosis (Table 2). Children with AKI had higher white blood cell and absolute neutrophil counts. Although there was no difference in circulating parasite density in children based on AKI status, children with AKI had higher levels of plasma HRP-2 as a measure of parasite biomass (adjusted *p* < 0·05) (Table 2). AKI is defined based on differences in kidney filtration, which may not reflect structural injury to the kidney. A high-risk NGAL result (NGAL ≥300 ng/mL) was associated with unresolved AKI at 24 h (69·1%) compared to children with complete AKI recovery (42·9%) (OR, 7·00 95% CI 4·16 to 11·76, *p* < 0·0.001, not shown) and children who died in-hospital (79·1%) compared to children who survived (38·6%) (OR, 6·02 95% CI 2·83 to 12·81) (Figure 2, Supplementary Table S4).

**Table 2:** Changes in laboratory measures on admission based on AKI status and severity.

	No AKI *n* = 327)	AKI (*n* = 271)	P value	Stage 1 AKI (*n* = 143)	Stage 2 AKI (*n* = 60)	Stage 3 AKI (*n* = 68)	P trend
Chemistries and blood gas							
Creatinine, mg/dL	0·30 (0·25, 0·34)	0·50 (0·43, 0·64)	<0·0001*	0·43 (0·39, 0·48)	0·59 (0·53, 0·64)	0·99 (0·74, 1·45)	<0·0001*
WHO renal impairment^1^, n (%)	0 (0·0)	6 (2·2)	0·008*	0 (0·0)	0 (0·0)	6 (8·8)	<0·001*
Blood urea nitrogen	9·0 (5·9, 12·9)	20·0 (12·0, 31·9)	<0·0001*	14·8 (9·0, 23·0)	21·0 (14·8, 30·0)	44·0 (26·3, 69·3)	<0·0001*
> 20 mg/dL, n (%)	19 (5·9)	132 (48·7)	<0·0001*	45 (31·5)	31 (51·7)	56 (82·4)	<0·001*
> 80 mg/dL, n (%)	0 (0·0)	12 (4·3)	<0·0001*	2 (1·4)	0 (0·0)	10 (14·7)	<0·001*
Chloride, mmol/L	104 (101, 107)	106 (103, 111)	<0·0001*	106 (103, 110)	106 (102, 111)	107 (103, 112)	<0·0001*
Hyperkalemia^2^, n (%)	4 (1·3)	31 (12·1)	<0·0001*	7 (5·1)	10 (18·2)	14 (21·5)	<0·001*
Hyponatremia^3^, n (%)	14 (4·7)	32 (12·4)	0·001*	15 (11·0)	5 (9·1)	12 (18·2)	0·002*
Hypoglycemia^4^, n (%)	6 (1·9)	18 (6·7)	0·003*	7 (4·9)	6 (10·0)	5 (7·4)	0·005*
Total bilirubin, mg/dL	0·5 (0·2, 0·9)	0·9 (0·5, 1·7)	<0·0001*	0·7 (0·3, 1·5)	1·1 (0·5, 1·7)	1·0 (0·6, 2·9)	<0·0001*
LDH, U/L	598 (442, 851)	840 (555, 1641)	<0·0001*	709 (479, 1057)	781 (625, 1388)	1561 (823, 3058)	<0·0001*
Plasma albumin	3·4 (2·9, 3·8)	3·5 (3·1, 3·9)	0·0007*	3·6 (3·2, 4·0)	3·4 (3·0, 3·9)	3·5 (3·1, 3·8)	0·045
AST, U/L	52 (36, 83)	84 (49, 173)	<0·0001*	68 (45, 121)	79 (47, 139)	183 (81, 418)	<0·0001*
ALT, U/L	18 (13, 25)	24 (17, 38)	<0·0001*	22 (16, 33)	24 (18, 30)	37 (24, 65)	<0·0001*
Lactate, mmol/L	3·7 (2·0, 5·7)	5·4 (2·8, 11·8)	<0·0001*	4·7 (2·6, 8·2)	5·9 (3·3, 13·2)	8·0 (2·8, 13·5)	<0·0001*
Base excess, mmol/L	−6 (−9, −3)	−12 (−18, −8)	<0·0001*	−11 (−16, −7)	−10 (−17, −7)	−16 (−21, −10)	<0·0001*
Bicarbonate, mmol/L	17·8 (15·8, 20·1)	13·2 (8·7, 16·5)	<0·0001*	13·9 (10·6, 16·7)	14·9 (8·0, 17·0)	9·8 (6·0, 15·0)	<0·0001*
pH	7·45 (7·39, 7·52)	7·38 (7·27, 7·45)	<0·0001*	7·39 (7·31, 7·47)	7·34 (7·24, 7·43)	7·35 (7·19, 7·43)	<0·0001*
Acidosis^5^, n (%)	87 (26·6)	189 (70·0)	<0·001*	94 (66·2)	39 (65·0)	56 (82·4)	<0·001*
Severe acidosis^5^, n (%)	8 (2·6)	58 (22·2)	<0·001*	24 (17·5)	14 (24·6)	20 (29·9)	<0·001*
Hematology							
WBC, x10^3^/uL	11·2 (8·2, 17·4)	13·7 (9·2, 22·5)	0·0002*	21·1 (8·6, 19·4)	12·5 (9·5, 20·1)	18·5 (10·6, 31·7)	<0·0001*
Neutrophil count, x10^3^/uL	5·7 (3·8, 8·6)	7·9 (5·0, 12·4)	<0·0001*	7·1 (4·6, 10·9)	7·5 (5·6, 11·5)	12·0 (6·3, 15·9)	<0·0001*
Eosinophil count, x10^3^/uL	0·02 (0·01, 0·10)	0·02 (0·01, 0·08)	0·294	0·01 (0·01, 0·05)	0·02 (0·01, 0·12)	0·05 (0·01, 0·11)	0·604
Platelet count, x10^3^/uL	116 (67, 217)	111 (56, 216)	0·544	117 (56, 221)	94 (47, 158)	129 (58, 275)	0·737
Hemoglobin, g/dL	5·6 (3·9, 8·4)	5·9 (3·4, 8·9)	0·532	6·3 (3·3, 9·5)	5·9 (3·9, 8·0)	5·0 (3·0, 7·4)	0·092
Microbiology							
Parasite density, /uL	8190 (0, 121,215)	17,228 (136, 164,844)	0·0965	27,515 (687, 164,844)	24,377 (924, 313,955)	2686 (0, 76,968)	0·677
Plasma HRP-2, ng/mL	2028 (262, 4709)	2643 (522, 6886)	0·031*	2245 (266, 6641)	4095 (1699, 8389)	1913 (373, 7304)	0·032*
HIV-1, n (%)	9 (2·8)	4 (1·5)	0·400	2 (1·4)	1 (1·7)	1 (1·5)	0·911
Bacteremia, n (%)	11/315 (3·5)	11/269 (4·1)	0·456	4 (2·8)	3 (5·0)	4 (6·0)	0·557

Data presented as median (interquartile range) or n (%).

1 World Health Organization (WHO) criteria for renal impairment is defined as a creatinine > 3 mg/dL.

2 Hyperkalemia defined as a serum potassium ≥ 6 mmol/L on admission.

3 Hyponatremia defined as a sodium < 130 mmol/L on admission.

4 Hypoglycemia defined as a glucose < 2·2 mmol/L on admission.

5 Acidosis defined as a base excess < –8 mmol/L, bicarbonate < 15 mmol/L if base excess was missing or a lactate > 5 mmol/L if bicarbonate and base excess were missing, severe acidosis defined as a bicarbonate < 8 mmol/L Adjusted estimates significant following adjustment for 29 multiple comparisons are indicated with an *.

### AKI recovery at 1 month and persistent kidney injury in survivors

**Figure 3:**
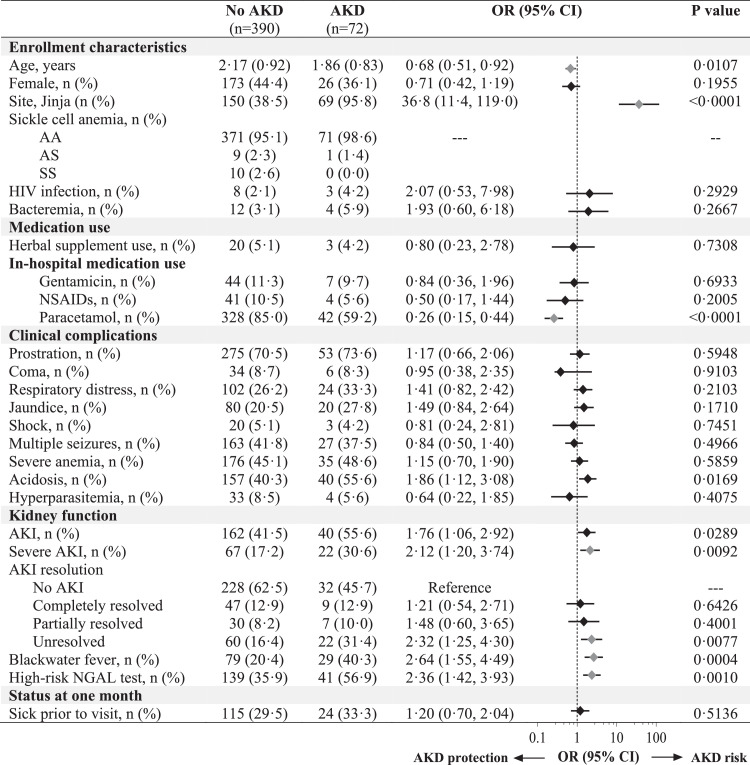
*Forest plot evaluating predictors of AKD in severe malaria survivors*. Forest plot evaluating risk factors for AKD at 1-month follow-up with the unadjusted odds ratio (OR) and 95% CI generated using logistic regression. Use of acetaminophen in hospital was associated with a reduced odds of AKD at 1 month follow-up while children from Jinja, with severe AKI on admission, unresolved AKI at 24 h, a history of blackwater fever and a high-risk NGAL test had an increased odds of AKD. Predictors significant following adjustment for 27 comparisons are indicated in pink.

The prevalence of kidney disease in community children was 6·8% compared to 15·6% in severe malaria survivors at 1-month follow-up (Supplementary Table S2). The prevalence of AKD at 1-month was highest in children with severe AKI at 24 h (36·8%) (not shown). Severe malaria was associated with a 2·54-fold increased odds of kidney disease compared to community children (95% CI 1·19 to 5·43) (not shown). Among children with severe malaria, AKI exposure was a risk factor for AKD at 1-month (OR, 1·76 95% CI 1·06 to 2·92) (Figure 3). The risk of AKD was highest in Jinja and among children with severe AKI, unresolved AKI at 24 h, a high-risk NGAL test, and in children with a history of blackwater fever (adjusted *p* < 0·05). Children who were treated with paracetamol during hospitalization had 74% reduced odds of AKD at 1-month follow-up (95% CI 0·15 to 0·44) (Figure 3).

NGAL levels at 1-month follow-up were significantly lower than on admission, and children with AKD had higher NGAL levels compared to children without AKD (*p* < 0·0001) (Figure 2).

### AKI-related elevated BUN is associated with mortality, duration of hospitalization, and neurodisability

AKI was present in 37/43 of admission deaths (86·1%). Mortality increased across AKI stages ranging from 1·8% in children without AKI to 7·7%, 13·3%, and 26·5% across AKI stages 1, 2 and 3 respectively (*p* < 0·0001, non-parametric test of trend, not shown). Severe metabolic disturbances were common in children with AKI and increased with increasing AKI severity (Table 2). Among children who died, 72·1% died within the first 24 h of hospitalization (not shown). In children with AKI, 27·3% of study participants had indications for kidney support therapy based on laboratory findings on admission (bicarbonate < 8 mmol/L, 22.2%; potassium ≥ 7.1 mmol/L, 8.2%; or BUN ≥ 100 mg/dL, 3.3%). The number of participants with biochemical abnormalities refractory to medical management is unknown as serial biochemical monitoring was conducted as clinically indicated, and not captured on study forms. None of the children with an indication for kidney support therapy were able to receive dialysis. Children with AKI were hospitalized longer than children without AKI (median 4·0 days vs. 3·0 days, *p* = 0·019). Using linear regression, unresolved AKI at 24 h (adjusted β +1.21 days, 95% CI 0·76 to 1·65, *p* < 0·0001) and elevated BUN (adjusted β +0·69 days, 95% CI 0·29 to 1·09, *p* = 0·0008) were both risk factors for prolonged hospitalization adjusting for age, sex, and site (Supplementary Table S5).

AKI was a risk factor for neurologic deficits on discharge (OR, 2·43, 95% CI 1·47 to 4·03) with 8·2% of children without AKI having neurologic deficits on discharge compared to 12·9% in children with complete AKI recovery, 17·1% in children with partial AKI recovery, and 28·9% in children with unresolved AKI (*p* < 0·001, Supplementary Table S6). Unresolved AKI and elevated BUN were independent risk factors for neurologic deficits at discharge adjusting for age, sex, and site (unresolved AKI, aOR 3·71, 95% CI 1·91 to 7·21; elevated BUN, aOR 2·84, 95% CI 1·66 to 4·87) (Supplementary Table S6). Among the 83 children with coma over hospitalization, 3 (3·6%) had BUN levels (> 80 mg/dL) consistent with a diagnosis of uremic encephalopathy (not shown).

### AKI and AKD interact to drive high post-discharge mortality

AKI was associated with increased post-discharge mortality, with 6·8% of children with AKI dying compared to 2·2% in children without AKI (*p* = 0·007, Supplementary Table S3). The presence of AKD at follow-up was also associated with increased post-discharge mortality, with 9·7% of children with AKD dying after 12 months of follow-up compared to 2·6% of children without AKD (*p* = 0·006, Supplemental Table S3). In children where AKD was confirmed as a post-AKI state, mortality was 17·5% compared to 3·7% in children with AKD with no recorded AKI exposure during hospitalization (Supplementary Table S3).

## Discussion

In this prospective multi-site study of Ugandan children with severe malaria, 45·3% of children had AKI. Consistent with previous studies, AKI was a risk factor for mortality, prolonged hospitalization, and neurologic deficits,^4^ with the risk highest in children with persistent AKI at 24 h. Malaria-associated AKI is likely heterogenous and multi-factorial. Histopathologic findings in malaria include acute tubular necrosis, interstitial nephritis and/or glomerulonephritis and may involve kidney-toxic medications, oxidative stress, endothelial dysfunction, immune-mediated damage, inflammation, and heme-toxicity.^4^^,^^17^ Among survivors, 16% had AKD at 1-month follow-up. Risk factors for AKD included AKI, severe AKI, unresolved AKI at 24 h, a high-risk NGAL value, and blackwater fever. AKD was associated with high post-discharge mortality. There were site-specific differences in the prevalence of both AKI and AKD, suggesting considerable heterogeneity in kidney disease within and between populations.

Early and sustained kidney recovery following AKI is associated with improved survival.^8^^,^^18^ In this study, 54% of children with community-acquired AKI rapidly recovered, with 63/193 (33%) of AKI recovering completely by 24 h suggesting that AKI is amenable to reversal with appropriate supportive care (Supplemental Table S2). However, a subset of children had persistent and severe AKI at 24 h follow-up which may occur in the context of high sequestered parasite biomass, where kidney perfusion cannot be restored with volume resuscitation, high concentrations of systemic inflammatory mediators,^18^ or structural injury to the kidney. Nephrotoxins, including herbal supplements and nephrotoxic medications, as well as the release of endogenous nephrotoxins from hemolysis (e.g., cell-free hemoglobin), may contribute to kidney injury.

In this study, 21·5% of children had persistent AKI at 24 h, with 11·3% of AKI classified as severe, representing a substantial burden of AKI following initial stabilization. Persistent AKI, typically defined based on AKI recovery at 48 h, is a risk factor for non-recovery, and long-term sequelae.^19^ More frequent creatinine assessments over hospitalization are needed to differentiate between transient AKI associated with rapid and sustained reversal and persistent AKI or relapsing AKI associated with AKD. In the present cohort, AKI was a risk factor for AKD. Children with severe or persistent AKI or evidence of tubular injury on admission had the highest risk of AKD, consistent with previous studies (reviewed in^8^). Children in sub-Saharan Africa have a high risk of mortality following hospital discharge that often equals, or exceeds, in-hospital mortality.^20^ In the present study, we demonstrate that children with AKD following AKI are at the highest risk of post-discharge mortality. To reduce post-discharge mortality, children surviving severe malaria at risk of persistent kidney disease should receive counselling to reduce exposure to nephrotoxins, education to understand the risk of repeated AKI episodes and CKD progression, and be prioritized for clinical follow up to assess CKD.

The identification of elevated BUN as a risk factor for prolonged hospitalization and neurologic deficits at discharge is novel in the context of malaria-associated AKI. AKI is a risk factor for long-term cognitive and behavioral problems^4^ and disabilities.^5^ In the context of malaria, AKI is associated with retinopathy, endothelial activation, blood-brain-barrier dysfunction, and increases in markers of axonal injury.^6^^,^^21^^,^^22^ Uremic toxins strongly induce endothelial dysfunction, accumulate within both the cerebrospinal fluid and brain tissue leading to neuroinflammation and oxidative stress, and have been implicated in cognitive impairment in CKD.^23^ Therefore, these findings raise questions on whether reducing exposure to neurotoxic nitrogenous waste products in the blood through expanded access to kidney support therapy may reduce long-term neurocognitive impairment following severe malaria.

As a large multi-site study assessing AKI in African children with severe malaria using KDIGO guidelines, we identified key site-related differences in the frequency of AKI and AKD. Children from Jinja were more likely to have AKI compared to children from Kampala. Herbal supplement use was more common in Jinja (6·7%) than Kampala (2·7%) and was identified as a risk factor for AKI, although the specific medications taken are unknown (Supplementary Table S2). Children from Jinja were more likely to have blackwater fever, which is a poorly understood complication of emerging clinical importance in Eastern Uganda associated with malaria infection.^24^ In the present study, blackwater fever was associated with an increased risk of AKD. Mechanistically, severe intravascular hemolysis in blackwater fever may drive direct heme-mediated tubular injury and hemeprotein-induced oxidative stress^25^^,^^26^ and maladaptive kidney repair, particularly if there is ongoing hemolysis. Rhabdomyolysis has also been reported in malaria^27^ and may contribute to pigment-induced AKI. The precise mechanisms responsible for the association between blackwater fever and progression from AKI to AKD warrant further investigation. In the present study, paracetamol administration during hospitalization was associated with reduced AKD, which is consistent with reports that paracetamol is kidney-protective by preventing oxidative stress-mediated kidney injury from endogenous nephrotoxins (e.g., hemoglobin, myoglobin).^28^^,^^29^ An ongoing randomized clinical trial evaluating paracetamol as adjunctive therapy in children with severe malaria may provide insight into whether paracetamol improves kidney recovery and repair (ClinicalTrials.gov, identifier: NCT04251351).

This study has several strengths, including recruitment of community children to estimate the burden of kidney disease in the study population,^16^ rigorous assessment of clinical outcomes, and active follow-up over one year to evaluate the impact of kidney injury on both in-hospital and post-discharge mortality. In addition, we assessed functional and structural markers of kidney injury in relation to kidney recovery and survival and present evidence of persistent functional impairment and structural injury at 1-month follow-up. The multi-site design enabled us to assess differences in AKI incidence, etiology, and post-AKI kidney recovery at two geographically and culturally distinct, but close, sites. This study adds to a growing list of prospective studies in LMIC, demonstrating that resource limitations need not be a barrier to conducting large-scale prospective clinical studies.

As this study was conducted before AKI was recognized as an important complication in children with severe malaria, there were limitations in defining AKI. Urine output was not assessed to define AKI, which may have led to the underestimation of AKI and imprecision in AKI staging.^3^ AKI recovery was assessed on stored samples collected at 24 h rather than 48 h as indicated in the KDIGO guidelines as a blood draw was not performed at 48 h. Further, incomplete creatinine data at 24 h may affect the reliability of our estimates on AKI progression and recovery and lead to detection or attrition bias. Children enrolled in the study did not have known creatinine measures prior to hospitalization so baseline creatinine was estimated using previously validated population approaches, and this may have led to misclassification of CKD as AKI or AKD over follow-up. As children with more severe AKI died, future studies assessing kidney recovery and CKD should take into consideration censoring due to death as a competing risk. As urine was not collected, we were unable to assess the etiology of blackwater fever, which was based on parental report of ‘coca cola’ coloured urine. Traditional herbal medicine use is widespread in sub-Saharan Africa− often used as first-line therapy in the context of illness− but frequently under-reported. In cases where herbal medicine use is reported, the identity and composition of compounds are often unknown. Studies are needed to understand the context of traditional medicine use in pediatric severe malaria and delineate whether kidney injury in some cases may be driven by, or exacerbated by, their use. Nephrotoxicity from traditional remedies, as with western medicines, may result from direct nephrotoxicity of compounds, be affected by contaminants or adulterants, be enhanced through incorrect preparation, storage, or use. In addition, medication nephrotoxicity may be impacted by disease- and/or individual- related risk factors as well as population genetics (e.g., glucose-6-phosphate dehydrogenase (G6PD) deficiency).

Although multi-organ dysfunction was common and children often presented with several features of severe malaria, the study enrolled children with selected manifestations of severe malaria which may affect the generalizability of the findings to less common severe malaria presentations (e.g., jaundice without other severe malaria criteria). Among children screened for inclusion, only 24 (3·7%) of hospitalized children were excluded because they did not have the specific severe malaria criteria outlined as inclusion criteria in this study. Screening was conducted seven days a week and 24 h a day to minimize selection bias. Children were included in the study based on symptoms consistent with malaria (history of fever, clinical signs of severe disease) and a diagnosis of malaria consistent with national guidelines (a positive blood smear or rapid diagnostic test). Clinical signs of severe malaria are non-specific and overlap with other causes of pediatric sepsis so we cannot rule out other causes of febrile illness with incidental malaria parasitemia. This reflects a common diagnostic dilemma, and it is important that clinicians use the diagnostic tools available in their setting when assessing and treating children hospitalized for fever. While the diagnosis of malaria in the study may have misclassified children with other febrile illnesses as having malaria, the use of a malaria definition that aligns with clinical guidelines increases the generalizability of the results. Additional studies are needed to understand disease-specific differences in AKI and AKD in cohorts enrolling children with acute febrile illnesses from varied causes. Within the study, the heterogeneity of findings at a sub-National regional level highlights a critical need for increased efforts to understand the burden and risk factors for AKI and persistent kidney disease in sub-Saharan Africa to make progress towards the International Society of Nephrology initiative to reduce preventable deaths due to AKI by 2025.^1^ Further, given limited data on AKI incidence and kidney recovery in children with severe malaria, additional studies are needed across sub-Saharan Africa to understand how differences in populations, malaria transmission intensity, antimalarial drug susceptibility, and other environmental and medicinal exposures impact AKI and kidney recovery. Standardized approaches to define AKI and kidney recovery across LMIC settings are needed.

AKI is an important clinical complication in severe malaria associated with prolonged hospitalization, neurologic deficits, persistent kidney disease, and mortality. For more than two decades, there has been debate as to whether patients are dying “from” or just “with” AKI. Although the preponderance of the pediatric AKI literature demonstrates a strong, independent association between Stage 2 AKI and poor outcomes, we recognize these data are associative. However, based on striking differences in pediatric AKI-related mortality in sub-Saharan Africa with 34% mortality overall and rising to 73% when kidney support therapy was indicated but not received,^30^ AKI is likely a causal mediator in severe malaria-associated mortality. Therefore, malaria associated AKI is a worthy target for the 0 by 25 AKI initiative.^1^ Further demonstration of causality will require interventions in at-risk patients prior to the development of Stage 2 AKI to show a survival benefit. Among AKI survivors, the immediate post-discharge period represents a critical window for clinical interventions to improve long-term renal recovery. Additional work is needed to define pathways of maladaptive repair following severe malaria-associated AKI and identify therapies that could target the initial recovery period to support adaptive kidney repair. Among AKI survivors that develop CKD, early diagnosis to ensure appropriate follow-up of high-risk patients and early initiation of therapies to manage hypertension and reduce CKD progression has the potential to avert even more preventable deaths.

## Declaration of interests

Dr. John reports grants from NIH during the conduct of the study. The other authors declare no competing interests.

## Appendix Supplementary materials

Image, application 1

Image, application 2
