# Supplementary material for: Acute kidney injury, persistent kidney disease, and post-discharge morbidity and mortality in severe malaria in children: A prospective cohort study
Source: eClinicalMedicine. 2022 Feb 12;44:101292. doi: 10.1016/j.eclinm.2022.101292 (PMC8850340; doi:10.1016/j.eclinm.2022.101292)
Supplement: Supplementary file 1 [file mmc1.docx]

**Supplement to:**

Namazzi R et al., Acute kidney injury, persistent kidney disease, and post-discharge morbidity and mortality in severe malaria in children: A prospective cohort study. *EClinicalMedicine*

**Contents**

[Table S1. Characteristics of study participants 2](#_Toc93039941)

[Table S2. Prevalence of kidney disease in children with severe malaria by study site 3](#_Toc93039942)

[Table S3. Risk factors for AKI by site 4](#_Toc93039943)

[Table S4. Relationship between kidney function and mortality 5](#_Toc93039944)

[Table S5. Relationship between kidney function and the duration of hospitalization 6](#_Toc93039945)

[Table S6. Relationship between kidney function and neurologic deficits at discharge 7](#_Toc93039946)

# **Table S1. Characteristics of study participants**

|  | **Combined Cohort** | **Kampala** | **Jinja** | **P value** |
| --- | --- | --- | --- | --- |
| **All participants** | **(n=716)** | **(n=396)** | **(n=320)** |  |
| Age in years, mean (SD) | 2.1 (0.9) | 2.2 (0.9) | 2.1 (0.9) | 0.036 |
| Sex, % Female | 316 (44.1) | 182 (46.0) | 134 (41.9) | 0.274 |
| **Severe malaria** | **(n=598)** | **(n=330)** | **(n=268)** |  |
| **Demographics** |  |  |  |  |
| Age in years, mean (SD) | 2.1 (0.9) | 2.2 (0.9) | 2.0 (0.9) | 0.005 |
| Sex, n (%) Female | 261 (43.7) | 147 (44.6) | 114 (42.5) | 0.622 |
| Reported herbal medication use, n (%) | 27 (4.5) | 9 (2.7) | 18 (6.7) | 0.019 |
| **Laboratory findings** |  |  |  |  |
| Parasite density, parasites/uL | 13075 (0, 135483) | 8577 (0, 114467) | 18430 (0, 164496) | 0.249 |
| Plasma HRP-2, ng/mL | 2362 (358, 5910) | 2450 (411, 6738) | 2176 (346, 5363) | 0.364 |
| Culture positive bacterial infections | 22/584 (3.8) | 12/325 (3.7) | 10/259 (3.9) | 0.915 |

Data presented median (IQR) unless otherwise indicated

Data analyzed using Student’s t-test or Wilcoxon rank sum test for continuous measures and Pearson’s Chi-square test for sex

# **Table S2. Prevalence of kidney disease in children with severe malaria by study site**

|  | **Combined Cohort** | **Kampala** | **Jinja** | **P value** |
| --- | --- | --- | --- | --- |
| **AKI over Hospitalization** | **(n=598)** | **(n=330)** | **(n=268)** |  |
| Any AKI, n (%) | 271 (45·3) | 117 (35·5) | 154 (57·5) | <0·001 |
| Maximum Stage, n (%)  Stage 1  Stage 2  Stage 3 | 143 (52·8)  60 (22·1)  68 (25·1) | 67 (57·3)  19 (16·2)  31 (26·5) | 76 (49·4)  41 (26·6)  37 (24·0) | 0·123 |
| Severe AKI, n (%) | 128 (21·4) | 50 (15·2) | 78 (29·1) | <0·001 |
| **Admission** | **(n=598)** | **(n=330)** | **(n=268)** |  |
| AKI^1^**,** n (%) | 263 (44·0) | 111 (33·6) | 152 (56·7) | <0·001 |
| AKI severity^2^**,** n (%)  Stage 1  Stage 2  Stage 3 | 146 (55·5)  65 (24·7)  52 (19·8) | 67 (60·4)  21 (18·9)  23 (20·7) | 79 (52·0)  44 (29·0)  29 (19·1) | 0·173 |
| Severe AKI^3^, n (%) | 117 (19·6) | 44 (13·3) | 73 (27·2) | <0·001 |
| **24 hours** | **(n=452)** | **(n=220)** | **(n=232)** |  |
| AKI^4^**,** n (%) | 97 (21·5) | 37 (16·8) | 60 (25·9) | 0·019 |
| AKI severity^2^**,** n (%)  Stage 1  Stage 2  Stage 3 | 46 (47·4)  14 (14·4)  37 (38·1) | 19 (51·4)  3 (8·1)  15 (40·5) | 27 (45·0)  11 (18·3)  22 (36·7) | 0·379 |
| Severe AKI^3^, n (%) | 51 (11·3) | 18 (8·2) | 33 (14·2) | 0·042 |
| Incident AKI^5^**,** n (%) | 8/259 (3·1) | 6/154 (3·9) | 2/105 (1·9) | 0·479 |
| Worsening AKI^6^**,** n (%) | 18/193 (9·3) | 8/66 (12·1) | 10/127 (7·9) | 0·336 |
| Improving AKI^7^**,** n (%) | 35/193 (18·1) | 9/66 (13·6) | 26/127 (20·5) | 0·242 |
| AKI resolution^8^**,** n (%)  Unresolved  Partial  Complete  No AKI | 97 (21·5)  41 (9·1)  63 (13·9)  251 (55·5) | 37 (16·8)  17 (7·7)  18 (8·2)  148 (67·3) | 60 (25·9)  24 (10·3)  45 (19·4)  103 (44·4) | <0·001 |
| **One-month follow-up** | **(n=462)** | **(n=243)** | **(n=219)** |  |
| AKD, n (%) | 72 (15·6) | 3 (1·2) | 69 (31·5) | <0·001 |

Differences in frequencies by site evaluated using Pearson’s Chi-square or Fisher’s exact test, as appropriate

^1^ Acute kidney injury defined using the KDIGO guidelines (1·5-fold increase in SCr from estimated baseline)

^2^ AKI staged as follows: Stage 1 (1·5-1·9x increase in SCr from estimated baseline), Stage 2 (2·0-2·9x increase in SCr from estimated baseline), Stage 3 (≥3·0 increase in SCr from estimated baseline or SCr ≥4·0mg/dL or eGFR<35mL/min per 1·73m^2^)

^3^Severe AKI defined as Stage 2 or Stage 3 AKI

^4^AKI defined using the KDIGO guidelines (1·5x fold increase in SCr from estimated baseline or 0·3mg/dL increase from admission)

^5^Incident AKI defined as AKI present at 24 hours follow-up in children without AKI on admission (n=259)

^6^ Worsening AKI defined as a higher AKI stage at 24 hours follow-up in children compared to admission in children with AKI on admission (n=193)

^7^ Improving AKI defined as AKI with a lower stage at 24 hours follow-up compared to admission (n=193)

^8^AKI was defined as unresolved if the participant met KDIGO AKI criteria at 24 hours, partial if AKI was present on admission and creatinine is within 50% of estimated baseline, and complete if creatinine is within 15% of estimated baseline in children with AKI on admission (n=193)

^9^Acute kidney disease (AKD) was defined as a 1·5-fold increase in creatinine over estimated baseline or an eGFR<90mL/min per 1·73m^2^ using the Bedside Schwartz equation

# **Table S3. Risk factors for AKI by site**

|  | **Kampala** | | | **Jinja** | | |
| --- | --- | --- | --- | --- | --- | --- |
|  | **No AKI**  **(n=213)** | **AKI**  **(n=117)** | **P value** | **No AKI**  **(n=114)** | **AKI**  **(n=154)** | **P value** |
| **Demographic Characteristics** |  |  |  |  |  |  |
| Age, years | 2·3 (0·9) | 2·1 (0·9) | 0·097 | 2·0 (0·9) | 2·0 (0·9) | 0·396 |
| Female, n (%) | 91 (42·7) | 56 (47·9) | 0·369 | 52 (45·6) | 62 (40·3) | 0·381 |
| Weight-for-age z score | -1·1 (1·1) | -1·0 (1·2) | 0·608 | -1·1 (1·0) | -1·1 (1·0) | 0·590 |
| Height-for-age z score | -1·2 (1·4) | -1·0 (1·3) | 0·147 | -1·2 (1·2) | -1·0 (1·3) | 0·216 |
| Weight-for-height z score | -0·6 (1·1) | -0·7 (1·3) | 0·475 | -0·7 (1·2) | -0·7 (1·1) | 0·747 |
| Duration of fever, days | 4·3 (2·9) | 3·5 (1·9) | 0·013 | 3·5 (2·0) | 2·8 (1·4) | 0·0008 |
| Sickle cell anemia, n (%)  HbAA  HbAS  HbSS | 203 (95·3)  3 (1·4)  7 (3·3) | 112 (95·7)  2 (1·7)  3 (2·6) | 0·916 | 108 (94·7)  3 (2·6)  3 (2·6) | 148 (96·1)  5 (3·3)  1 (0·7) | 0·402 |
| **Medication History** |  |  |  |  |  |  |
| Anti-malarial, n (%) | 117 (57·1) | 69 (60·0) | 0·611 | 81 (71·1) | 97 (63·0) | 0·167 |
| Artesunate, n (%) | 43 (21·0) | 22 (19·1) | 0·694 | 10 (8·8) | 16 (10·4) | 0·658 |
| Antibiotic, n (%) | 60 (29·3) | 39 (33·9) | 0·388 | 40 (35·1) | 64 (41·6) | 0·283 |
| Gentamicin, n (%) | 10 (4·9) | 6 (5·2) | 0·894 | 3 (2·6) | 5 (3·3) | 1·000 |
| NSAIDs, n (%) | 26 (12·2) | 16 (13·7) | 0·702 | 7 (6·1) | 3 (2·0) | 0·103 |
| Herbal medication use, n (%) | 4 (1·9) | 5 (4·3) | 0·289 | 3 (2·6) | 15 (9·7) | 0·025 |
| **Admission characteristics** |  |  |  |  |  |  |
| Temperature, °C | 37·6 (1·2) | 37·5 (1·3) | 0·557 | 37·8 (1·2) | 37·9 (1·3) | 0·588 |
| Tachycardia, n (%) | 113 (53·1) | 60 (51·3) | 0·758 | 62 (54·4) | 105 (68·2) | 0·021 |
| Tachypnea, n (%) | 145 (68·1) | 87 (74·4) | 0·232 | 75 (65·8) | 120 (77·9) | 0·027 |
| Shock, n (%) | 15 (7·1) | 13 (11·4) | 0·192 | 3 (2·7) | 18 (11·9) | 0·006 |
| Coma, n (%) | 18 (8·5) | 16 (13·7) | 0·139 | 11 (9·7) | 28 (18·2) | 0·050 |
| Multiple seizures, n (%) | 96 (45·1) | 50 (42·7) | 0·683 | 47 (41·2) | 58 (37·7) | 0·554 |
| Retinopathy, n (%) | 20/184 (10·9) | 29/97 (29·9) | <0·0001 | 4 (5·3) | 9 (11·0) | 0·251 |
| Respiratory distress, n (%) | 28 (13·2) | 39 (33·3) | <0·0001 | 28 (24·6) | 80 (52·0) | <0·001 |
| Prostration, n (%) | 127 (59·6) | 93 (79·5) | <0·0001 | 80 (70·2) | 129 (83·8) | 0·008 |
| Jaundice, n (%) | 38 (17·8) | 32 (27·6) | 0·039 | 20 (17·5) | 47 (30·5) | 0·015 |
| Blackwater fever, n (%) | 31 (14·7) | 27 (23·3) | 0·052 | 25 (21·9) | 58 (37·7) | 0·006 |
| Severe anemia, n (%) | 94 (44·3) | 49 (41·9) | 0·667 | 38 (33·3) | 63 (41·5) | 0·177 |
| Vomiting, n (%) | 100 (47·0) | 61 (52·1) | 0·367 | 53 (46·5) | 101 (65·6) | 0·002 |
| Diarrhea, n (%) | 29 (13·6) | 23 (19·7) | 0·149 | 28 (24·6) | 35 (22·7) | 0·726 |
| **Comorbid diagnoses** |  |  |  |  |  |  |
| Gastroenteritis, n (%) | 33 (15·6) | 26 (22·2) | 0·137 | 10 (8·8) | 19 (12·3) | 0·353 |
| Pneumonia, n (%) | 7 (3·3) | 9 (7·7) | 0·078 | 12 (10·5) | 16 (10·4) | 0·971 |

Data presented as mean (SD) or n (%)

Differences in means evaluated by Student’s t-test

Differences in frequencies by site evaluated using Pearson’s Chi-square or Fisher’s exact test, as appropriate

# **Table S4. Relationship between kidney function and mortality**

|  | **Deaths** | **OR (95% CI)** | **P value** | **aOR (95% CI)** | **P value** |
| --- | --- | --- | --- | --- | --- |
| **In-hospital mortality** | **(n=43)** |  |  |  |  |
| AKI  No AKI  AKI | 6/327 (1·8)  37/271 (13·7) | Reference  8·46 (3·51, 20·37) | ---  <0·0001 | Reference  8·57 (3·51, 20·89) | ---  <0·0001 |
| High risk NGAL test  No high risk NGAL test  High risk NGAL test | 9/347 (2·6)  34/246 (13·8) | Reference  6·02 (2·83, 12·81) | ---  <0·0001 | Reference  5·74 (2·67, 12·31) | ---  <0·0001 |
| **Post-discharge mortality** | **(n=23)** |  |  |  |  |
| AKI  No AKI  AKI | 7/321 (2·2)  16/234 (6·8) | Reference  3·29 (1·33, 8·14) | ---  0·010 | Reference  2·94 (1·16, 7·43) | ---  0·023 |
| AKI resolution  No AKI  Complete  Partial  Unresolved | 7/250 (2·8)  4 /62 (6·5)  3/41 (7·3)  7/90 (7·8) | Reference  2·39 (0·68, 8·45)  2·74 (0·68, 11·06)  2·93 (1·00, 8·59) | ---  0·175  0·157  0·051 | Reference  2·23 (0·61, 8·16)  2·65 (0·64, 10·96)  2·71 (0·90, 8·16) | ---  0·245  0·178  0·076 |
| AKD  No AKD  AKD | 10/390 (2·6)  7/72 (9·7) | Reference  2·09 (1·50, 11·13) | ---  0·006 | Reference  5.77· (1·51, 22·10) | ---  0·127 |
| AKI and AKD  No AKI, No AKD  AKI, No AKD  No AKI, AKD  AKI, AKD | 4/228 (1·8)  6/162 (3·7)  0/32 (0·0)  7/40 (17·5) | Reference  2·15 (0·60, 7·76)  ---^1^  7·77 (3·30, 42·80) | ---  0·241  ---  <0·0001 | Reference  2·54 (0·69, 9·40)  ---^1^  22·52 (4·02, 126·35) | ---  0·161  ---  <0·0001 |

Logistic regression used with mortality (in-hospital or post-discharge) as the dependent variable and kidney function as the independent variable

Models adjusted for age, sex, and site

^1^ No deaths occurred in this group, so an estimate was unavailable

# **Table S5. Relationship between kidney function and the duration of hospitalization**

|  | **Beta (95% CI)** | **P value** | **aBeta (95% CI)** | **P value** |
| --- | --- | --- | --- | --- |
| AKI^1^ | 0·45 (0·10, 0·81) | 0·012 | 0·56 (0·20, 0·92) | 0·002 |
| AKI resolution^1^  No AKI  Complete  Partial  Unresolved | Reference  0·06 (-0·45, 0·58)  0·43 (-0·19, 1·05)  1·14 (0·70, 1·58) | ---  0·806  0·171  <0·0001 | Reference  0·16 (-0·37, 0·69)  0·48 (-0·14, 1·11)  1·21 (0·76, 1·65) | ---  0·557  0·128  <0·0001 |
| Elevated BUN^2^ | 0·66 (0·26, 1·06) | 0·001 | 0·69 (0·29, 1·09) | 0·0008 |

Linear regression used with duration of hospitalization as the dependent variable and kidney function as the independent variable

Models adjusted for age, sex, and site

# **Table S6. Relationship between kidney function and neurologic deficits at discharge**

|  | **Neurologic deficits (n=73)** | **OR (95% CI)** | **P value** | **aOR (95% CI)** | **P value** |
| --- | --- | --- | --- | --- | --- |
| AKI^1^  No AKI  AKI | 28/314 (8·9)  45/234 (19·2) | Reference  2·43 (1·47, 4·03) | ---  0·0006 | Reference  1·99 (1·18, 3·35) | ---  0·010 |
| AKI resolution^1^  No AKI  Complete  Partial  Unresolved | 20/245 (8·2)  8/62 (12·9)  7/34 (17·1)  26/90 (28·9) | Reference  1·67 (0·70, 3·99)  2·32 (0·91, 5·89)  4·57 (2·40, 8·72) | ---  0·251  0·078  <0·0001 | Reference  1·23 (0·50, 3·01)  1·96 (0·75, 5·10)  3·71 (1·91, 7·21) | ---  0·650  0·168  0·0001 |
| Elevated BUN^2^  No elevated BUN  Elevated BUN | 43/426 (10·1)  29/120 (24·2) | Reference  2·84 (1·68, 4·79) | ---  0·0001 | Reference  2·84 (1·66, 4·87) | ---  0·0001 |

Logistic regression used with neurologic deficits as the dependent variable and kidney function as the independent variable

Models adjusted for age, sex, and site
