# Supplementary material for: Acute kidney injury, persistent kidney disease, and post-discharge morbidity and mortality in severe malaria in children: A prospective cohort study
Source: eClinicalMedicine. 2022 Feb 12;44:101292. doi: 10.1016/j.eclinm.2022.101292 (PMC8850340; doi:10.1016/j.eclinm.2022.101292)
Supplement: Supplementary file 2 [file mmc2.docx]

STROBE Statement—checklist of items that should be included in reports of observational studies

|  | Item No. | Recommendation | Page  No. | Relevant text from manuscript |
| --- | --- | --- | --- | --- |
| **Title and abstract** | 1 | (*a*) Indicate the study’s design with a commonly used term in the title or the abstract | Page 2 | 598 children with severe malaria and 118 community children aged six months to four years were prospectively recruited from two sites in Uganda and followed for 12-months to assess survival. |
|  |  | (*b*) Provide in the abstract an informative and balanced summary of what was done and what was found | Page 2 | The Kidney Disease: Improving Global Outcomes (KDIGO) criteria were used to define AKI (admission, 24 hours) and AKD (one-month follow-up). False Discovery Rate was used to adjust for multiple comparisons. |
| Introduction | | | |  |
| Background/rationale | 2 | Explain the scientific background and rationale for the investigation being reported | Page 4 | " Although AKI is increasingly recognized in children with severe malaria (reviewed in3), the burden of malaria-associated AKI in LMICs and its impact on lifelong health outcomes, including CKD, remains poorly understood.”  Because of the rising global burden of CKD and its associated strain on health systems^11^, as well as the historical under-representation of sub-Saharan African in global AKI research^4^, there is a critical need to define AKI recovery and predictors of AKD in malaria. |
| Objectives | 3 | State specific objectives, including any prespecified hypotheses | Page 4 | We hypothesize that children in sub-Saharan Africa with malaria-associated AKI will have kidney dysfunction persist as AKD. To test this hypothesis, we defined the epidemiology of community-acquired AKI, AKI recovery, and AKD in a prospective multi-site observational study of Ugandan children with severe malaria followed for one year. We evaluated risk factors for AKI and AKD to define the impact of persistent kidney disease on morbidity and mortality in the context of severe malaria. |
| Methods | | | |  |
| Study design | 4 | Present key elements of study design early in the paper | Page 5 | Between 2014 and 2017, 600 children were enrolled in a prospective cohort study of children with severe malaria at two sites in Uganda to evaluate the impact of the five most common forms of severe malaria on neurocognition at 12-months follow-up in children <5 years of age. |
| Setting | 5 | Describe the setting, locations, and relevant dates, including periods of recruitment, exposure, follow-up, and data collection |  | Children were recruited from Mulago National Referral and Teaching Hospital (MNRH) in Kampala in Central Uganda and Jinja Regional Referral Hospital (JRRH) in Jinja in Eastern Uganda (Figure 1). MNRH is the main National Referral Hospital for the country, located in the Kampala metropolitan area |
| Participants | 6 | (*a*) *Cohort study*—Give the eligibility criteria, and the sources and methods of selection of participants. Describe methods of follow-up  *Case-control study*—Give the eligibility criteria, and the sources and methods of case ascertainment and control selection. Give the rationale for the choice of cases and controls |  | Children presenting to hospital with a history of fever were screened by study teams working at the participating sites seven days a week, 24 hours a day. Inclusion criteria included children age six months to four years of age with a positive rapid diagnostic test for Plasmodium falciparum histidine-rich protein-2 (HRP-2) or direct visualization of parasites by Giemsa microscopy who required hospitalization for prostration; severe anemia, hemoglobin<5g/dL; two or more seizures in 24 hours; respiratory distress; or coma13. The definition of malaria using either rapid diagnostic test or microscopy aligns with the Uganda Clinical Guidelines on malaria diagnosis making the results more generalizable. Exclusion criteria included known chronic illness requiring medical care, known developmental delay, or history of coma, head trauma, and prior hospitalization for malnutrition. None of the children had a known history of kidney disease. Comatose children with lumbar puncture findings suggestive of another cause of coma were excluded. For controls, 120 community children from the nuclear family, extended family, or household area of the children with severe malaria were enrolled and used to assess the prevalence of kidney disease in the population and validate approaches to estimate baseline creatinine. Additional exclusion criteria for the controls included an illness requiring medical care within the previous four weeks, a major medical or neurologic abnormality at screening physical examination, or active illness. |
|  |  | (*b*) *Cohort study*—For matched studies, give matching criteria and number of exposed and unexposed  *Case-control study*—For matched studies, give matching criteria and the number of controls per case |  | N/A |
| Variables | 7 | Clearly define all outcomes, exposures, predictors, potential confounders, and effect modifiers. Give diagnostic criteria, if applicable | Page 5 | The primary exposure was acute kidney injury, and the primary outcome was kidney recovery at one-month follow-up (AKD). Secondary exposures included AKI severity, persistent AKI, and a high-risk NGAL test. Secondary outcomes included in-hospital mortality, duration of hospitalization, the presence of neurologic deficits at discharge, and post-discharge 12-month mortality. |
| Data sources/ measurement | 8* | For each variable of interest, give sources of data and details of methods of assessment (measurement). Describe comparability of assessment methods if there is more than one group | Page 6  Page 8 | “Elevated BUN was defined as a BUN ≥20mg/dL^13^. Blackwater fever was defined based on parental report of tea or ‘coca-cola’ coloured urine, which has a reported agreement of 80% with urine dipsticks for hemoglobin6. Shock was defined as a capillary refill time >3 seconds or a lower limb temperature gradient.”  “Acute kidney injury (AKI) was defined using the Kidney Disease: Improving Global Outcomes (KDIGO) criteria based on a 1·5-fold increase in creatinine from baseline or a 0·3mg/dL increase in creatinine within 48 hours15. Urine output was not assessed as it is not a standard clinical practice in Uganda. AKI was staged as follows: stage 1, 1·5-1·9-fold increase in creatinine over baseline; stage 2, 2·0-2·9-fold increase over baseline; stage 3, ≥3·0-fold increase over baseline. AKI was classified as severe if it was stage 2 or 33. Baseline SCr was estimated using a height-independent approach assuming a GFR of 120mL/min per 1·73m2 as described16. The height-independent approach to estimate baseline SCr was validated as the best approach to estimate baseline creatinine using two independent cohorts of Ugandan community children and outperformed other approaches16. Creatinine was measured on admission, at 24 hours, and one-month follow-up. AKI was defined as incident if AKI was not present on admission and was diagnosed at 24 hours. AKI recovery was assessed at 24 hours and was defined as unrecovered if AKI was still present, partial if creatinine was within 50% of estimated baseline creatinine, or complete if the creatinine returned to within 15% of estimated baseline creatinine.  Acute kidney disease (AKD) was defined in children with severe malaria if there was a 1·5-fold increase in creatinine over estimated baseline or an eGFR<90mL/min per 1·73m2 at one-month follow-up. The same definition was applied to community children and classified as kidney disease.” |
| Bias | 9 | Describe any efforts to address potential sources of bias | Page 16 | “Screening was conducted seven days a week and 24 hours a day to minimize selection bias.”  “Children were included in the study based on symptoms consistent with malaria (history of fever, clinical signs of severe disease) and a diagnosis of malaria consistent with national guidelines (a positive blood smear or rapid diagnostic test). Clinical signs of severe malaria are non-specific and overlap with other causes of pediatric sepsis so we cannot rule out other causes of febrile illness with incidental malaria parasitemia. This reflects a common diagnostic dilemma, and it is important that clinicians use the diagnostic tools available in their setting when assessing and treating children hospitalized for fever. While the diagnosis of malaria in the study may have misclassified children with other febrile illnesses as having malaria, the use of a malaria definition that aligns with clinical guidelines increases the generalizability of the results.” |
| Study size | 10 | Explain how the study size was arrived at | Page 5 | “We conducted a power calculation assuming a mortality rate of 8%^13^, 95% retention at one-month follow-up, and varied the AKI prevalence from 35-50%. At a fixed alpha of 0.05, we will have between 83-87% power to detect a two-fold increase in AKD in children with AKI during hospitalization assuming a baseline AKD prevalence of 10% in children without AKI” |

Continued on next page

|  | | | **Item No.** | **Recommendation** | **Page No.** | | | | **Relevant text from manuscript** | |
| --- | --- | --- | --- | --- | --- | --- | --- | --- | --- | --- |
| Quantitative variables | | 11 | | Explain how quantitative variables were handled in the analyses. If applicable, describe which groupings were chosen and why | | Page 8-9 | | Statistical analysis section | |  |
| Statistical methods | | 12 | | (*a*) Describe all statistical methods, including those used to control for confounding | | Page 8-9 | | Statistical analysis section | |  |
|  |  |  |  | (*b*) Describe any methods used to examine subgroups and interactions | | Table S2 | | Site stratified differences in variables associated with AKI are presented in Table S2 | |  |
|  |  |  |  | (*c*) Explain how missing data were addressed | | Page 8 | | Analyses were conducted on all participants with AKI and AKD assessed as outlined in Figure 1. Data on age, sex, and site were complete for all participants. | |  |
|  |  |  |  | (*d*) *Cohort study*—If applicable, explain how loss to follow-up was addressed  *Case-control study*—If applicable, explain how matching of cases and controls was addressed  *Cross-sectional study*—If applicable, describe analytical methods taking account of sampling strategy | | Figure 1 | |  | |  |
|  |  |  |  | (*e*) Describe any sensitivity analyses | | Page 10 | |  | |  |
| Results | | | | | | | | | |  |
| Participants | | 13* | | (a) Report numbers of individuals at each stage of study—eg numbers potentially eligible, examined for eligibility, confirmed eligible, included in the study, completing follow-up, and analysed | | Figure 1 | |  | |  |
|  |  |  |  | (b) Give reasons for non-participation at each stage | | Figure 1 | |  | |  |
|  |  |  |  | (c) Consider use of a flow diagram | | Figure 1 | |  | |  |
| Descriptive data | | 14* | | (a) Give characteristics of study participants (eg demographic, clinical, social) and information on exposures and potential confounders | | Table 1 | | Five hundred and ninety-eight children with severe malaria enrolled in the study **(Figure 1**). The mean (SD) age of children was 2·1 (0·9) years with 43·7% of children female. Enrollment across sites was comparable with 44·8% of children enrolled from Jinja and 55·2% from Kampala. | |  |
|  |  |  |  | (b) Indicate number of participants with missing data for each variable of interest | | Figure 1 | |  | |  |
|  |  |  |  | (c) *Cohort study*—Summarise follow-up time (eg, average and total amount) | | Page 9 | | The mean (SD) duration of follow-up for participants was 0·87 (0·25) person-years. | |  |
| Outcome data | | 15* | | *Cohort study*—Report numbers of outcome events or summary measures over time | | Figure 1  Page 9-12 | |  | |  |
|  |  |  |  | *Case-control study—*Report numbers in each exposure category, or summary measures of exposure | |  | |  | |  |
|  |  |  |  | *Cross-sectional study—*Report numbers of outcome events or summary measures | |  | |  | |  |
| Main results | | 16 | | (*a*) Give unadjusted estimates and, if applicable, confounder-adjusted estimates and their precision (eg, 95% confidence interval). Make clear which confounders were adjusted for and why they were included | | Table 1  Figure 3 | |  | |  |
|  |  |  |  | (*b*) Report category boundaries when continuous variables were categorized | | Table 2 | |  | |  |
|  |  |  |  | (*c*) If relevant, consider translating estimates of relative risk into absolute risk for a meaningful time period | |  | |  | |  |
| Other analyses | 17 | | Report other analyses done—eg analyses of subgroups and interactions, and sensitivity analyses | | |  |  | | |  |
| Discussion | | | | | | | | | |  |
| Key results | 18 | | Summarise key results with reference to study objectives | | | Page 12 |  | | |  |
| Limitations | 19 | | Discuss limitations of the study, taking into account sources of potential bias or imprecision. Discuss both direction and magnitude of any potential bias | | | Page 15 |  | | |  |
| Interpretation | 20 | | Give a cautious overall interpretation of results considering objectives, limitations, multiplicity of analyses, results from similar studies, and other relevant evidence | | | Page 15  Page 16-17 |  | | |  |
| Generalisability | 21 | | Discuss the generalisability (external validity) of the study results | | | Page 15-16 |  | | |  |
| Other information | | |  | | | | | | |  |
| Funding | 22 | | Give the source of funding and the role of the funders for the present study and, if applicable, for the original study on which the present article is based | | | Abstract  Page 18 |  | | |  |

*Give information separately for cases and controls in case-control studies and, if applicable, for exposed and unexposed groups in cohort and cross-sectional studies.

**Note:** An Explanation and Elaboration article discusses each checklist item and gives methodological background and published examples of transparent reporting. The STROBE checklist is best used in conjunction with this article (freely available on the Web sites of PLoS Medicine at http://www.plosmedicine.org/, Annals of Internal Medicine at http://www.annals.org/, and Epidemiology at http://www.epidem.com/). Information on the STROBE Initiative is available at www.strobe-statement.org.
